# Genenames.org: the HGNC and PGNC resources in 2026

**DOI:** 10.1093/nar/gkaf1229

**Published:** 2025-11-25

**Authors:** Ruth L Seal, Bryony Braschi, Kristian Gray, James McClay, Susan Tweedie, Elspeth A Bruford

**Affiliations:** HUGO Gene Nomenclature Committee, Department of Haematology, University of Cambridge, School of Clinical Medicine, Cambridge CB2 0PT, UK; HUGO Gene Nomenclature Committee, Department of Haematology, University of Cambridge, School of Clinical Medicine, Cambridge CB2 0PT, UK; HUGO Gene Nomenclature Committee, Department of Haematology, University of Cambridge, School of Clinical Medicine, Cambridge CB2 0PT, UK; HUGO Gene Nomenclature Committee, Department of Haematology, University of Cambridge, School of Clinical Medicine, Cambridge CB2 0PT, UK; HUGO Gene Nomenclature Committee, Department of Haematology, University of Cambridge, School of Clinical Medicine, Cambridge CB2 0PT, UK; HUGO Gene Nomenclature Committee, Department of Haematology, University of Cambridge, School of Clinical Medicine, Cambridge CB2 0PT, UK

## Abstract

The HUGO Gene Nomenclature Committee (HGNC), based at the University of Cambridge, approves unique symbols and descriptive names for human genes. The HGNC database currently contains over 44 400 approved gene symbols, over 19 250 of which represent protein-coding genes, ∼14 500 represent pseudogenes, and over 9500 represent non-coding RNA genes. The public website, www.genenames.org, displays all approved nomenclature within manually curated Symbol Reports and also displays related groups of genes in Gene Group Reports. In 2024, we formed the Plant Gene Nomenclature Committee (PGNC), which has been approving gene symbols and names for the tree species *Populus trichocarpa*. All approved plant gene nomenclature is displayed on the new website plant.genenames.org. Here, we review updates to the HGNC project and introduce the PGNC project.

## Graphical Abstract

**Graphical Abstract:**
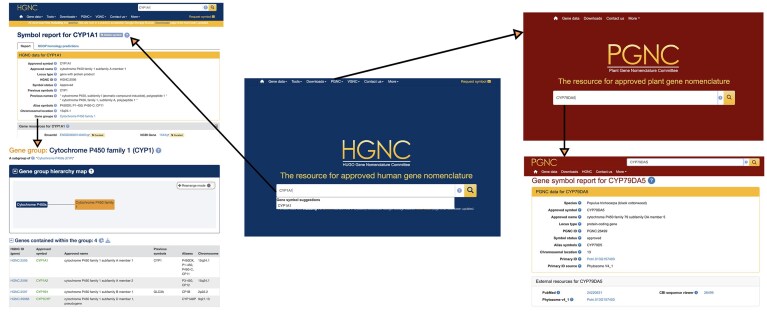

## Introduction

The HUGO Gene Nomenclature Committee (HGNC) aims to approve a unique and standardized gene name and symbol for every human gene and is one of the longest-running resources that delivers biological standards for the scientific community. In 2024, the team relocated from the European Molecular Biology Laboratory-European Bioinformatics Institute (EMBL-EBI) at the Wellcome Genome Campus in Hinxton to the Cambridge Biomedical Campus, with all staff members now employed by the University of Cambridge. The essential role we play has been recognized by our inclusion in the Global Biodata Coalition [1] as a Global Core Biodata Resource (GCBR) and by ELIXIR as an ELIXIR-UK Core Data Resource [2]. We are also a recommended resource on FAIRsharing [3]. Despite these acknowledgements that the HGNC is a crucial resource for biomedical science, since our last database publication [4], we have suffered a reduction in staff and a period of several months where some current team members were on secondment due to a gap in the start of new funding.

In 2024, the HGNC was awarded 5 years of NIH funding, and we are currently focusing on the following key aims. First, we will continue standardized naming and curation of human protein-coding genes, RNA genes, and pseudogenes with input from the scientific research community, our panel of specialist advisors, and annotators within the Ensembl/GENCODE [5] and NCBI RefSeq [6] resources. Second, we plan to expand into transcript naming in collaboration with the biocuration community, including input from the UniProt [7] resource. We will continue coordinating gene naming in vertebrates, in collaboration with the model organism databases (the Mouse Genome Database (MGD) [8], the Rat Genome Database (RGD) [9], the *Xenopus* database Xenbase [10], the Zebrafish Information Network (ZFIN) [11], and the Chicken Gene Nomenclature Consortium (CGNC) database [12]), as well as naming genes in further key vertebrate species as part of our Vertebrate Gene Nomenclature Committee (VGNC project), which is described in a separate publication [13] and not presented in this database report. We will engage external stakeholders in both the research and clinical communities, to encourage awareness of and compliance with approved gene nomenclature - the first activity for this aim has been to engage with genetic counselors, described later in this paper.

All HGNC data are presented on our website, https://www.genenames.org. Each HGNC-approved gene symbol has a corresponding descriptive gene name and a unique HGNC ID. HGNC Symbol Reports on our website display this nomenclature in a core data section, along with numerous links to other biomedical resources, gene orthology information, and relevant publications. Our website also provides a number of useful tools, including a SOLR-based search, HCOP: HGNC’s Comparison of Orthology Predictions, a Multi-Symbol Checker, and a BioMart service; see Table 1 for a description of each tool. All of our data is freely accessible and can be downloaded using several different methods, including a REST service, a Custom Downloads tool, and via text or JSON files.

**Table 1.** A guide to the main tools available on genenames.org

Tool Name	Purpose	URL
**Search**	SOLR-based website search tool that supports both basic and advanced, field-specific searches. Provides search suggestions after 2 or more characters are entered. Results can be filtered by report type, gene entry status, locus type, or symbol report tag. Search results can be downloaded in text or JSON format.	https://www.genenames.org/tools/search/
**HCOP**	HGNC Orthology Predictions search tool. Provides aggregated orthology predictions between human and 19 different species, including all major non-plant model organisms, from 12 independent orthology resources. A bulk download facility with precomputed files is available, and each individual search result can be downloaded as a text file.	https://www.genenames.org/tools/hcop/
**Multi-Symbol Checker**	A tool that compares one or many search terms against all approved, previous, withdrawn, and alias symbols in the HGNC database. Supports file uploads or entering terms in a search box. Returns a sortable table of results containing a row for each match, which can be downloaded in CSV format.	https://www.genenames.org/tools/multi-symbol-checker/
**BioMart**	A tool for building complex queries to create custom data sets. The genenames.org BioMart hosts two separate HGNC Marts: **Gene Mart** for gene data queries and **Family Mart** for gene group queries. Results can be downloaded as a text file.	https://biomart.genenames.org/

Here we describe updates made to the HGNC resource since our last report in 2023 [4]. In addition to our core NIH funding, we have expanded to include a new project funded by the U.S. Department of Energy to name genes in the tree model organism *Populus trichocarpa*, in collaboration with the Oak Ridge Center for Bioenergy Innovation. The resulting new plant gene nomenclature resource, https://plant.genenames.org, is described below.

## HGNC data

### New gene entries in genenames.org

**Figure 1.**
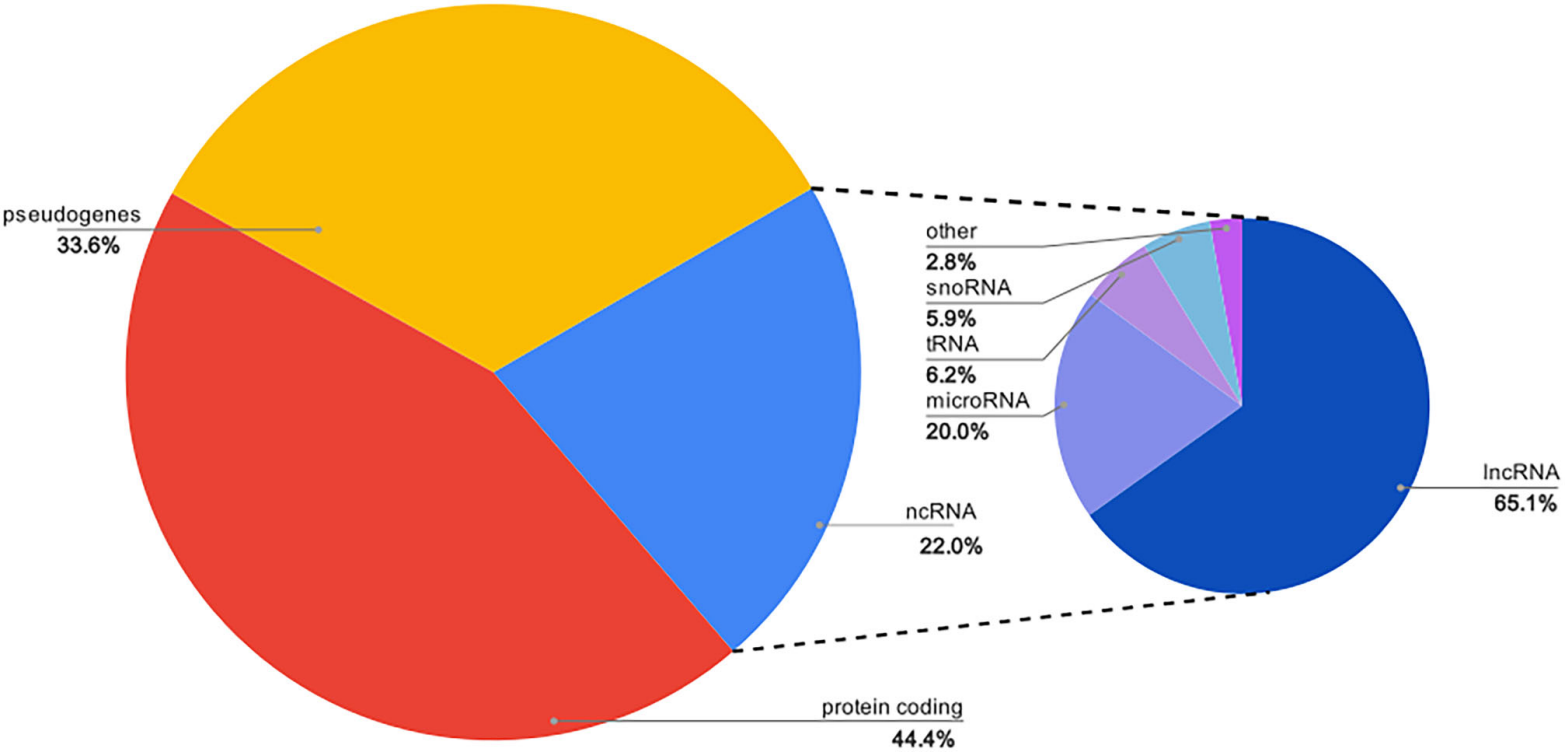
Pie chart showing the proportion of human genes named by the HGNC for each main locus type (protein coding, pseudogene, and non-coding (nc)RNA). Numbers of genes named per ncRNA subtype are shown in the breakout pie; subtypes shown are small nucleolar (sno)RNA, transfer (t)RNA, microRNA, and long non-coding (lnc)RNA. The “other” ncRNA category includes small nuclear RNA and ribosomal RNA.

The HGNC approves symbols for genes of several different biotypes, the main categories of which are protein-coding, non-coding RNA, and pseudogenes. See https://www.genenames.org/download/statistics-and-files for a full list of our biotypes and the associated numbers of genes with approved symbols. As of September 3, 2025, the HGNC website contains 44 476 genes in total with 19 294 protein coding genes, 14 603 pseudogenes and 9574 ncRNA genes—of which the largest sub-categories are long non-coding RNA (6236 genes), microRNA (1912 genes), transfer RNA (591 genes), and small nucleolar RNA (568 genes), see Fig. 1 for a chart displaying the proportion of named genes for these locus types.

The set of protein-coding genes with HGNC symbols, based on current manual gene annotation guidelines, is relatively complete. Most HGNC work on protein-coding genes focuses on renaming genes with placeholder symbols and evaluating the stability of existing gene symbols, as described later in this paper. There are some examples of “novel” protein-coding genes that have been recently described in publications and subsequently assigned approved nomenclature by the HGNC, including *MLDHR* (HGNC:55 481), which was identified by [14] as located within the 5′ UTR of the *PTEN* (HGNC:9588) gene. The authors published this gene as “MP31” for “micropeptide of 31 amino acids,” which does not fulfill HGNC guidelines due to the mention of the number of amino acid residues. Based on the functional information in [14], the HGNC approved the unique, searchable gene symbol *MLDHR* with the corresponding gene name “mitochondrial lactate dehydrogenase regulator.” A second example is *SMIM10L3* (HGNC:56 768), which was identified in [15] and is also an example of a protein-coding gene nested within the 5′ UTR of a separate, characterized protein-coding gene, *FAM220A* (HGNC:22 422). The authors of [15] identified the gene in both mouse and human and used the nomenclature “SAGSIN1” for “salivary gland specific protein 1″ based on its expression in mouse tissues. However, this gene has four human paralogs: *SMIM10* (HGNC:41 913), *SMIM10L1* (HGNC:49 847), *SMIM10L2A* (HGNC:34 499), and *SMIM10L2B* (HGNC:34 500), so the HGNC approved the gene symbol *SMIM10L3* to represent gene family membership.

The HGNC also names novel protein-coding genes that have no associated publications but have been identified by gene annotators. *RNF228* (ring finger protein 228, HGNC:55 809), a single-exon gene, was recently annotated based on support from phyloCSF [16] and Ribo-Seq data. *NPIPA9* (nuclear pore complex interacting protein family member A9, HGNC:41 984) is another recent example that had been missed previously due to readthrough annotation between this and a neighbouring gene. In these two cases, the HGNC was able to approve informative nomenclature based on membership of the two genes within separate gene families.

The HGNC is actively increasing its named pseudogene catalog, with 548 named since [4]. There are three main types of pseudogene: duplicated, also known as unprocessed pseudogenes, that are formed by duplicated genome sequence; retrotransposed, also known as processed pseudogenes, that are formed by reverse transcription of mRNA into genomic DNA; and unitary pseudogenes that are degraded copies of ancestral functional genes. Examples of duplicated pseudogenes named recently by the HGNC include *WDR5CP* (WD repeat domain 5C, pseudogene, HGNC:57 046) and *VOPP2P* (VOPP family member 2, pseudogene, HGNC:58 490). Processed pseudogenes are usually named relative to the parent gene from which they were retrotransposed; recently named examples include *MTMR9P1* (MTMR9 pseudogene 1, HGNC:56 931), *STMN3P1* (STMN3 pseudogene 1, HGNC:56 985), and *PHF10P2* (PHF10 pseudogene 2, HGNC:56 986). Unitary pseudogenes are named based on functional orthologs identified in other species; recent examples include RNF*4BP* (ring finger protein 4B, pseudogene, HGNC:58 117) and *PTPN11BP* (protein tyrosine phosphatase non-receptor type 11B, pseudogene, HGNC:58 112). We have recently created a new gene group for unitary pseudogenes (https://genenames.org/data/genegroup/#!/group/3418).

In terms of non-coding RNA gene naming, some of our gene categories, such as microRNA genes, are comparatively comprehensive. Most of our current work focuses on the expanding long non-coding RNA (lncRNA) field, with 795 lncRNA genes named since our last database report in 2023 [4]. LncRNAs are the only type of ncRNA where researchers may request a symbol based on an identified characteristic of the gene; recent examples of lncRNA genes named based on publications include *CYDAER* (cytoplasmic differentiation associated epidermal RNA, HGNC:40 594) based on [17]; *VILMIR* (virus inducible lncRNA modulator of interferon response, HGNC:56 888) based on [18] and *LNCRMP* (long non-coding RNA regulator of monocyte proliferation, HGNC:58 164) based on [19]. In some cases, the original published symbol is not suitable for approval by the HGNC; in these instances, we discuss alternatives with the authors of the respective paper. Examples include *DAGARR* (differentiation and growth arrest related lncRNA, HGNC:58 123), initially published as *DAGAR* [20], which was modified to improve its searchability in literature search engines and *CACSTL1* (chromosomal aberration CCDC6::RET stimulating lncRNA 1, HGNC:56 690), initially published as *CASTL1* [21], which was unsuitable because *CASTL1* could have been misinterpreted as representing a gene that was like the protein-coding *CAST* (HGNC:1515) gene in sequence.

The HGNC has a systematic method for naming annotated lncRNAs lacking published data. These are named based on their genomic location relative to protein-coding genes, see [22] for a full description. We have worked mostly on expanding our antisense lncRNA category (587 new entries since last database publication), where the lncRNA is named relative to a protein-coding gene on the opposite strand with the symbol suffix -AS e.g *RXFP1-AS1* (RXFP1 antisense RNA 1, HGNC:40 897).

### Marking genes as stable

The HGNC is committed to keeping the symbols of clinically relevant genes stable whenever possible to prevent confusion within clinical settings. The HGNC is a member of the Gene Curation Coalition (GenCC) [23], a project that focuses on standards for curation of genes that are causative of human disease. We are reviewing the nomenclature of genes in the GenCC database and adding the “stable” tag to the Symbol Reports on www.genenames.org of genes where we have evaluated the symbol and consider that it will not be changed in the future. While genes in the GenCC database are our priority, we may review and add the tag to other genes that we look at as part of our daily curation work.

**Figure 2.**
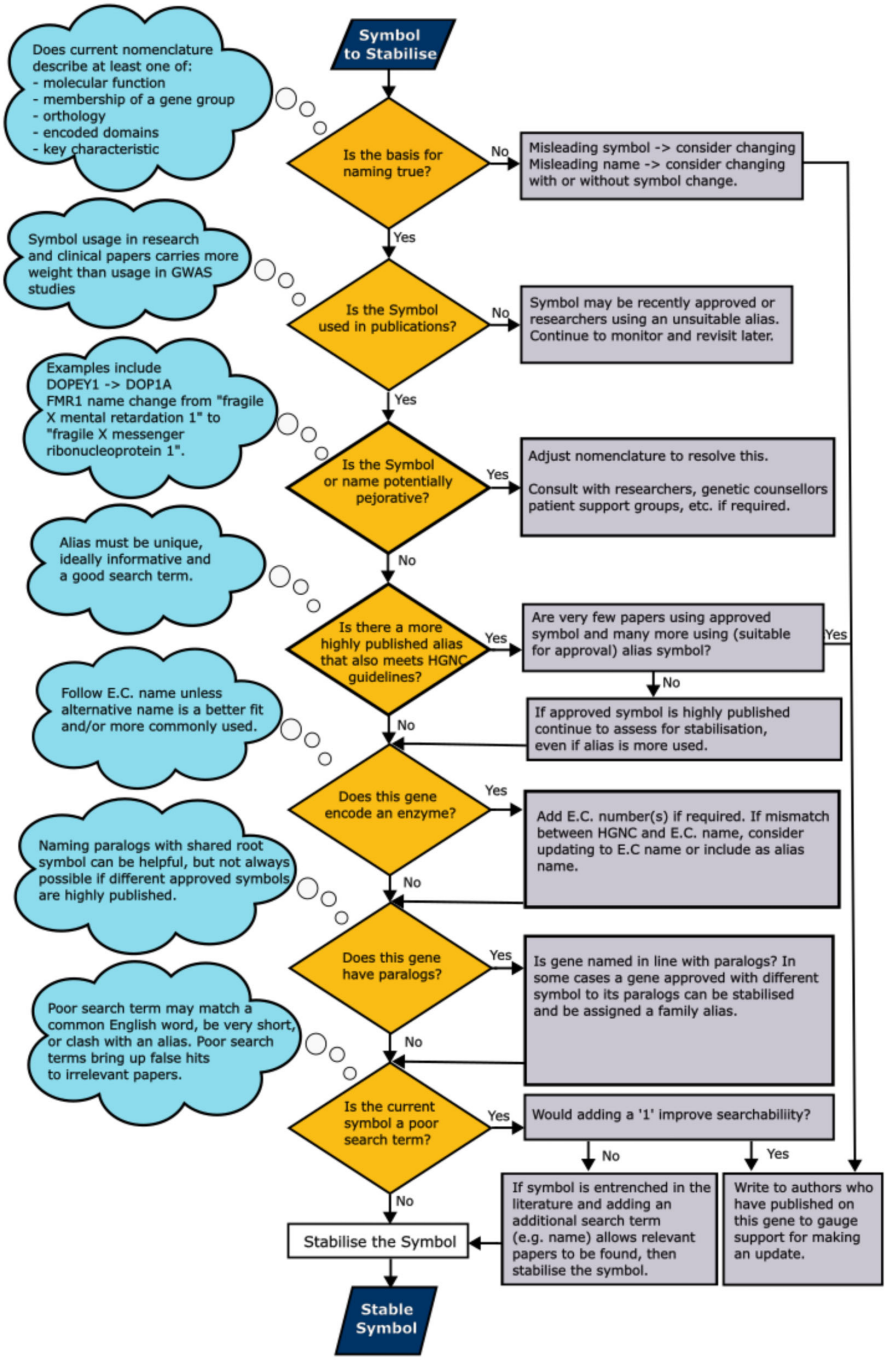
Flowchart of steps taken when considering gene symbols for stabilization.

Fig. 2 shows a flowchart of the symbol stabilization process. The first step involves checking that approved symbols are not misleading and cannot be interpreted as being pejorative or offensive. Gene symbols that fail this check are evaluated further to see if changes are appropriate; such changes are discussed with relevant researchers and clinical groups, and in some cases, with patient support groups. Gene symbols that pass the first part of the review are then checked for usage in literature *vs*. usage of symbol aliases, and are checked for searchability in literature engines and assessed for any potential problems in data handling. The second phase of the test is judged on a case-by-case basis, and only factors considered to be serious problems will result in a gene symbol change. A caveat to this is that genes with placeholder-type nomenclature may be updated in order to provide more informative, stable symbols for the future. For some genes, we are able to keep the gene symbol stable, but we update the gene name to be more descriptive. Gene name changes are less disruptive to the clinical community than symbol changes.

Since our last database report [4], we have marked 599 genes as stable, bringing us to a total of 3581 genes with this tag. For most of these stabilized genes, no nomenclature changes were made; examples are *DLAT* (HGNC:2896), *DLD* (HGNC:2898), *ATP6AP2* (HGNC:18 305), *BRWD3* (HGNC:17 342), *AIPL1* (HGNC:359), *DBT* (HGNC:2698), *ADK* (HGNC:257), *BACE1* (HGNC:933), and *CHM* (HGNC:1940). Examples where the symbol was not changed but the name was changed to be more informative are *EP300* (HGNC:3373), where the name was updated from “E1A binding protein p300” to “EP300 lysine acetyltransferase,” and *PHIP* (HGNC:15 673) where the name was updated from “pleckstrin homology domain interacting protein” to “PHIP subunit of CUL4-Ring ligase complex.” An example of a gene where the symbol was changed prior to the stable tag being added is *POPDC1* (HGNC:1152)—the previous symbol and name for this gene was *BVES* for “blood vessel epicardial substance”; this was misleading because the gene was originally isolated from the heart, but is not specifically expressed in blood vessels. The gene symbol *POPDC1* had already been published (e.g. [24, 25]) and the change made its nomenclature consistent with its family members *POPDC2* (HGNC:17 648) and *POPDC3* (HGNC:17 649).

In addition to 3524 protein-coding genes with the stable tag, we have 53 non-coding RNA genes marked with this tag. A proportion of these are from a review that we performed on the first set of non-coding genes included in the Matched Annotation from NCBI and EMBL-EBI (MANE) project [26]. The MANE project produces a set of representative transcripts for genes on the current reference human genome (GRCh38), where each transcript has an identical annotated sequence in the NCBI RefSeq [6] and Ensembl/GENCODE [5] gene sets; in the majority of cases, each gene has only one representative MANE transcript. We were able to add stable tags to the following well-published genes included in this dataset: *RNU12* (HGNC:19 380), *BACE1-AS* (HGNC:37 125), *MIAT* (HGNC:33 425), *IFNG-AS1* (HGNC:43 910), *ATXN8OS* (HGNC:10 561), *NR2F1-AS1* (HGNC:48 622), and *GAS5* (HGNC:16 355). All of these are lncRNA genes, except for *RNU12*, which encodes a small nuclear RNA and has been identified as causing CDAGS syndrome [27]. For some of the genes in the initial ncRNA MANE dataset, we had already added a stable tag, such as *RNU4-2* (HGNC:10 193) and *XIST* (HGNC:12 810), that had entries in the GenCC database. More recently, we added the stable tag to the *RNU2-2* (HGNC:10 152) gene following its identification as a causative gene for a neurodevelopmental disorder [28, 29]. Previously, this gene had been annotated as a pseudogene and approved as *RNU2-2P* (RNA, U2 small nuclear 2, pseudogene), but data in the papers and discussion with annotators resulted in the update of this gene to a small nuclear RNA gene.

### Renaming pre-existing gene entries

While the HGNC is committed to minimizing symbol changes, we do aim to update our “placeholder” symbols so that these genes have informative nomenclature that can be transferred across species. Our placeholder symbols include ‘**C%orf#’** where % represents the chromosome that the gene is located on, orf stands for ‘open reading frame’ and # is an iterative number; **FAM#** for “family with sequence similarity” where paralogous genes are named together in the absence of further information to reflect family membership; and **KIAA#** symbols that were approved for genes identified by the Kazusa cDNA sequencing project and could not be given a more informative symbol at the time of naming. Only 427 protein-coding genes with C%orf#s, FAM#s, and KIAA#s symbols remain.

We receive alerts from the PubMed database when new papers mention “C%orf#” genes, and we regularly search the https://www.biorxiv.org database for preprints that feature these symbols. Examples of recent changes to ‘C%orf#’ symbols based on pre-publication agreement with authors include HGNC:15 873 updated from *C20orf27* to *ADISSP* (adipose secreted signaling protein) based on [30]; HGNC:28 144 updated from *C9orf64* to *QNG1* (Q-nucleotide N-glycosylase 1) based on [31]; HGNC:26 730 updated from *C1orf127* to *CIROZ* (ciliated left-right organizer protein containing ZP-N domains) based on [32]. The paralogs HGNC:33 818, HGNC:34 242, and HGNC:27 938 were renamed together away from the shared root symbol FAM166 (family with sequence similarity 166) to the shared root symbol CIMIP2 (ciliary microtubule inner protein 2) based on [33], resulting in the approved symbols *CIMIP2A, CIMIP2B*, and *CIMIP2C*.

### Utilising AI/ML in gene naming

Pfam-N matches are the result of a deep learning method by Google DeepMind that was trained on Pfam data [34], which has identified matches between proteins and pre-existing Pfam domains that were not called by the Pfam database previously. We evaluated Pfam-N matches for proteins encoded by genes with HGNC placeholder symbols to check if this would allow us to approve more informative nomenclature based on protein domains. The following placeholder symbols were updated: HGNC:31 418 and HGNC:25 275 were updated from *C9orf131* and *C2orf16* to *SPATA31G1* (SPATA31 subfamily G member 1) and *SPATA31H1* (SPATA31 subfamily H member 1) based on a Pfam-N domain match to the PF14650 (SPATA31) domain in the encoded proteins, which is found in other SPATA31 family members. HGNC:23 378 was updated from *KIAA2026* to *BRD10* (bromodomain containing 10) based on a Pfam-N match to the PF00439 bromodomain, and HGNC:29 301 was updated from *KIAA1522* to *NHSL3* based on a Pfam-N match to the PF15273 (NHS-like protein) domain.

### Renames based on biotype

In addition to updating placeholder nomenclature, the HGNC makes symbol changes to reflect a change in gene biotype when necessary. Biotypes may change from pseudogene to protein-coding gene, e.g. HGNC:43 621 was updated from *SSU72P2* (SSU72 pseudogene 2) to *SSU72L2* (SSU72 like 2) and HGNC:43 611 was updated from *IGBP1P2* (immunoglobulin (CD79A) binding protein 1 pseudogene 2) to *IGBP1C* (IGBP1 family member C) and vice versa, e.g. HGNC:25 344 was changed from *C2orf83* (chromosome 2 open reading frame 83) to *SLC19A4P* (solute carrier family 19 member 4, pseudogene). Equally, biotypes can change from protein coding to lncRNA, e.g. HGNC:26 385 was changed from *C14orf178* (chromosome 14 open reading frame 178) to *SLIRP-OT1* (SLIRP overlapping transcript 1) and vice versa, e.g. HGNC:44 353 was updated from *LINC00672* (long intergenic non-protein coding RNA 672) to *LASP1NB* (LASP1 neighbor).

As can be seen from all of the above examples, when HGNC symbols change, the HGNC ID stays the same. Therefore, we recommend that all databases track approved symbols using HGNC IDs to stay up to date.

### Community outreach

A key aim of our current funding is to engage with external stakeholders, encouraging their compliance with using approved gene nomenclature. Ongoing HGNC outreach activities include publishing a blog (https://blog.genenames.org) and posting on Bluesky (hgnc.bsky.social). It is important that researchers and clinicians use or at least mention HGNC-approved symbols in their work in order to avoid confusion and improve data retrieval [35]. The HGNC will continue to engage with journal editors and publishers and to consult with researchers and clinicians to increase awareness of HGNC-approved nomenclature and to promote its usage. The HGNC is also actively reaching out to genetic counselors for the first time; genetic counseling is a constantly expanding field of undeniably increasing importance in the genomics era.

Genetic counselors need to be able to communicate with patients and their families clearly and effectively. A key part of their job is to explain genetic conditions to enable a patient or their carer to make an informed decision about whether to undergo genetic testing. They then help interpret test results and provide advice and support, depending on the outcome. It is vital that a counselor can show empathy towards and provide emotional support for their clients. It would be very unhelpful if a gene name or symbol were to be perceived as insulting or flippant by a patient or their family, or if the gene nomenclature were to seriously hinder rather than help communication in any way.

The HGNC curators put together a survey asking “Does how genes are named matter to genetic counselors and their patients?” and publicized this at the UK-based AGNC (Association of Genetic Nurses and Counsellors) conferences and via a targeted Research Survey E-Blast message sent to members of the US-based NSGC (National Society of Genetic Counselors). Feedback from this survey may help resolve a small number of particularly difficult cases, where researchers strongly feel their suggested symbols and gene names are appropriate for use, but the HGNC is hesitant to approve them as they could potentially be perceived as pejorative. The HGNC will look carefully at the survey responses and see how we can best resolve any issues that may be highlighted. The results of this survey will be fully discussed in an upcoming publication.

### New gene groups

In addition to Symbol Reports, www.genenames.org contains 1946 Gene Group reports. These groups may represent genes with a shared root symbol, shared homology, shared function, shared membership of a complex, or shared encoded protein domain. Within the last three years, we have added 248 new manually curated gene groups to this resource. Where applicable, we curate our gene groups into hierarchies to show relationships between groups—an example of a new gene group placed in a hierarchy is the ISWI complexes (https://www.genenames.org/data/genegroup/#!/group/3336) gene group, which has been placed beneath the ATP-dependent chromatin remodeling complexes parent group and has the subgroups: ACF complex, B-WICH chromatin-remodeling complex subunits, CERF complex, Chromatin accessibility complex, NoRC complex, NURF complex, RSF complex, and WICH complex.

The HGNC has a new collaboration with SciBite/Elsevier, who use our gene group data within their commercial software tools. SciBite has suggested some additional groups that will improve the coverage of our data, and is providing funding for these to be added. The initial focus has been on adding groups for genes that are drug targets, many of which are enzymes. As of September 3, 2025, we have added 68 new groups for this collaboration, including 36 for kinase families. We are also adding new aliases for gene groups based on SciBite’s internal vocabularies to improve searchability.

## HGNC website updates

### Migration from on-premises infrastructure to Google Cloud Platform

Since our move from EMBL-EBI, we have successfully completed a comprehensive migration of the HGNC infrastructure from traditional on-premises virtual machines (VMs) and physical load balancers to a modern cloud-native architecture on Google Cloud Platform. This transformation represents a significant modernization effort, moving from a traditional datacenter deployment to a fully managed cloud infrastructure while maintaining the critical functionality of the HGNC services. The migration provides automatic scaling, built-in redundancy, and simplified deployment workflows. We replaced our physical load balancer with Google’s global External HTTPS Load Balancer, which now handles SSL termination, provides DDoS protection through Cloud Armor security policies, and offers global traffic distribution capabilities. This cloud-native load balancing solution eliminates the need for hardware maintenance while providing superior performance and reliability.

We modernized our database infrastructure by migrating to fully managed Cloud SQL instances for both MySQL and PostgreSQL databases, removing the operational overhead of database administration, patching, and backups. The SOLR search infrastructure, previously running on VMs, has been reimplemented as a high-availability cluster using Google Compute Engine instances with persistent disks. This architecture maintains the flexibility of VM-based deployments while leveraging Google’s infrastructure for improved reliability.

The migration also introduced modern cloud-native capabilities that were not available in our traditional datacenter environment. These include automated resource scheduling to optimize costs, comprehensive monitoring and alerting through Google Cloud’s operations suite, Infrastructure as Code using Terraform for consistent and repeatable deployments, and enhanced security through IAM, Workload Identity, and Secret Manager. The new architecture leverages VPC networking with Cloud NAT for secure outbound connectivity, while Cloud Scheduler and Pub/Sub provide robust automation capabilities for scheduled tasks and event-driven workflows. All HGNC download files, including the archive files, are now in a publicly accessible Google Storage Bucket, which is available through our site at https://www.genenames.org/download/statistics-and-files.

## Introducing the PGNC website

Following on from our well-established VGNC project (vertebrate.genenames.org) [13], the HGNC has a new sister project, plant.genenames.org. The plant gene nomenclature committee (PGNC) team is a collaboration with colleagues at the Oak Ridge Center for Bioenergy Innovation. The project has started with approving nomenclature for the model organism *Populus trichocarpa*, a large deciduous tree also known as the black cottonwood, western balsam-poplar, or California poplar. This fast-growing, economically important species has a fully sequenced genome [36] and a substantial body of research literature. We recently published an opinion piece on the process of building nomenclature guidelines with community input for *P. trichocarpa* [37].

The primary focus is naming protein-coding genes that have been studied in *P. trichocarpa*, such as *BSPA* (bark storage protein A, PGNC:26 030) and *BSTR* (booster, PGNC:24 432). We are also naming well-conserved genes that are already consistently named across a wide range of species, such as *APC1* (anaphase promoting complex subunit 1, PGNC:17 255) and *CDC6* (cell division cycle 6, PGNC:2257). As with human and other vertebrate gene naming, we are engaging the help of specialist advisors to name complex gene families such as the cytochrome P450 (CYP) genes.

**Figure 3.**
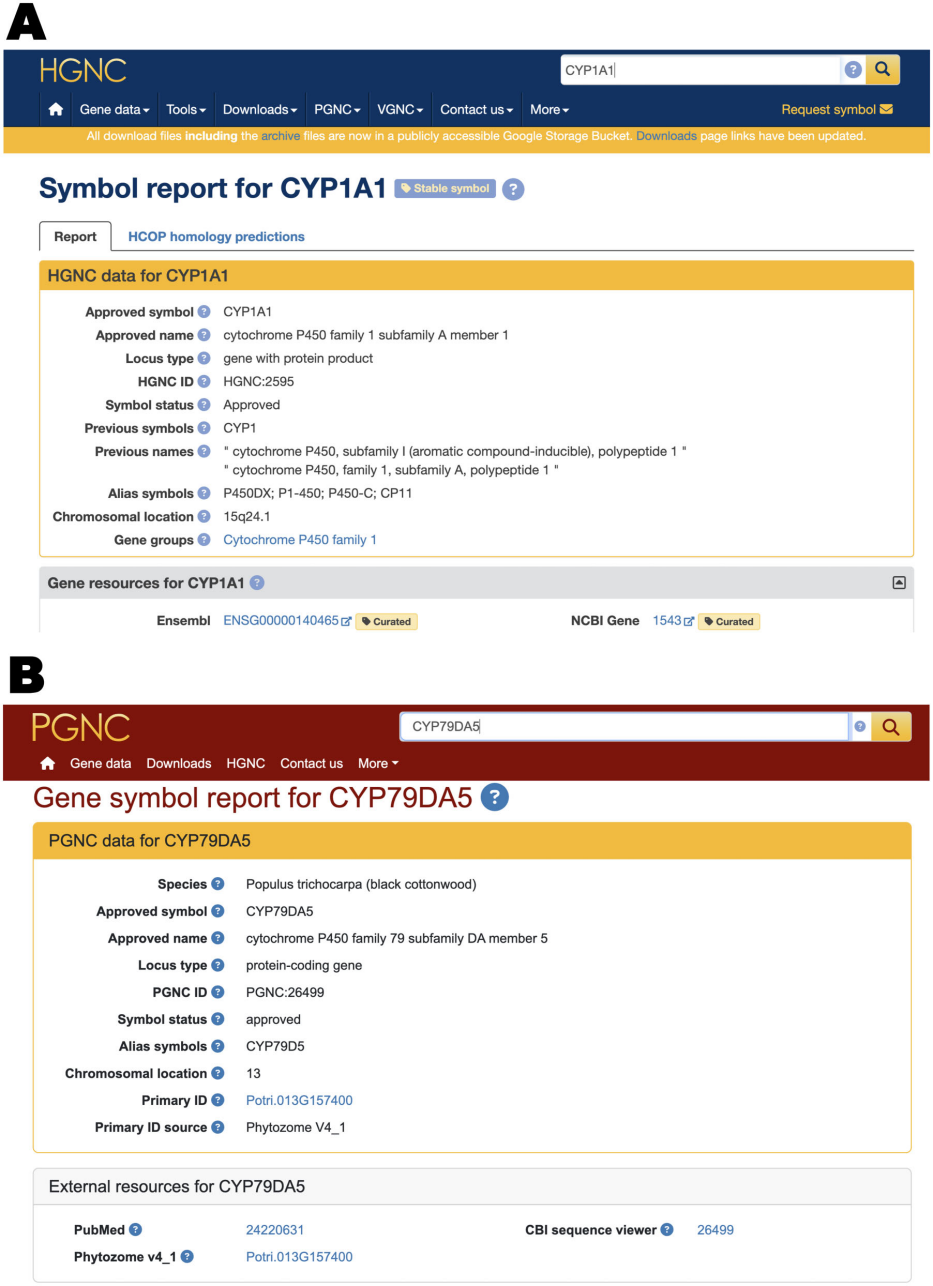
Screenshots showing the consistent look and feel for Symbol Reports of two genes from the cytochrome P450 gene family on www.genenames.org and plant.genenames.org. (**A**) Screenshot of the top part of the CYP1A1 Symbol Report, showing the Core Data box and top gene resources. Symbol Reports on www.genenames.org have the following further sections below this: Nucleotide resources, Protein resources, Orthologs, Specialist resources, Clinical Resources, Other resources, and References. Note the stable tag at the top of the Symbol Report, described elsewhere in this paper. (**B**) Screenshot of the CYP79DA5 Symbol Report on plant.genenames.org. Note the primary ID field in the core data section, which shows the source of the plant gene annotation.

All approved plant gene nomenclature is displayed on the plant.genenames.org website, which has a consistent look and feel to genenames.org - see the Symbol Report for *CYP1A1* (HGNC:2595) on genenames.org (Fig. 3A) and the Symbol Report for the cytochrome P450 family gene *CYP79DA5* (PGNC:26 499) on plant.genenames.org (Fig. 3B). The PGNC website can also be accessed directly from the PGNC tab on the genenames.org website. The PGNC website hosts a SOLR search, and each plant.genenames.org Symbol Report contains a unique PGNC ID, the name of the plant species, a gene symbol, gene name, and gene aliases, with links to the relevant NCBI Gene ID [6], UniProt protein ID [7], and, where available, a PubMed reference ID. One key difference from genenames.org Symbol Reports is the concept of the “primary ID,” which for *P. trichocarpa* is the Phytozome gene ID from the Phytozome database [38].

All PGNC database and website infrastructure is hosted on Google Cloud Platform. The stack is designed for scalability, maintainability, and ease of deployment, making it suitable for both development and production environments. The project uses a containerized microservices architecture orchestrated with Docker Compose, consisting of a backend PostgreSQL database, an Angular 19 + web application, Solr Client, and Apache Solr for searching, a Python Data Loader for data processing, and a NestJS RESTful API for programmatic access to gene data. An Nginx web server is used as a reverse proxy and load balancer. All plant gene data are also available to download in JSON format from https://github.com/PGNC-Plant-Gene-Nomenclature-Committee/Downloads.

## Future directions

The HGNC will continue with its aims of approving informative gene symbols for protein-coding genes, non-coding RNA genes, and pseudogenes, and increasing the number of Symbol Reports marked with the Stable tag. We will develop rules for automating the naming of well-annotated independently transcribed long non-coding RNA genes. We will continue to update the nomenclature of placeholder symbols as new information becomes available and will update the nomenclature of genes based on biotype changes where necessary. We will also expand our gene group resource.

The HGNC will actively collaborate with specialized biomedical projects such as IUPHAR [39], GenCC [23], HUGO [40], RNAcentral [41]; we also recently joined the Disease Naming Advisory Committee (DNAC) [42]. We hold regular meetings with our colleagues at the mouse and rat genomic nomenclature committees [8, 9] to discuss complex nomenclature issues across species. And we will continue to take part in discussions about the annotation and naming of new loci, in particular novel genes added to the MANE dataset [26], the sub-types of open reading frames found in the “dark proteome,” and any newly identified loci with the Human Pangenome Reference [43], as well as investigating the naming of transcripts in complex loci with the biocuration community. Finally, we will carry on our work to increase awareness and usage of approved gene nomenclature. We intend to publish the full results of our genetic counselor survey and to contact journals to request review of their nomenclature requirements.

The PGNC project will aim to expand on the curation of *P. trichocarpa* genes, focusing on well-published genes and gene families. If funding allows, this project could also be expanded to gene naming in other key plant model organisms that currently lack any nomenclature authority.

## Data Availability

HGNC services are freely available at https://www.genenames.org. PGNC services are freely available at https://plant.genenames.org/. PGNC external stack code is available at https://doi.org/10.5281/zenodo.17380839. The following code for the HGNC project is available: **HGNC-EDirect** (code that contains NCBI’s EDirect scripts and HGNC scripts that utilise NCBI EDirect): https://doi.org/10.5281/zenodo.17359848 **HGNC/europe-pmcentralizer** (code that fetches PubMed IDs and creates references for each valid PubMed ID from Europe PubMed Central): https://doi.org/10.5281/zenodo.17359626 **HGNC/get-gene-info** (code that retrieves HGNC gene symbol reports via a list of gene IDs): https://doi.org/10.5281/zenodo.17359674 **HGNC/hgnc-gene-family-mapper** (code that draws an HGNC gene group hierarchy map): https://doi.org/10.5281/zenodo.17359925 **HGNC/iHAG** (code that creates an interactive hierarchical acyclic graph for HGNC gene group hierarchy maps): https://doi.org/10.5281/zenodo.17359995 **HGNC/pfam-dom-draw** (code that takes an HGNC-approved symbol, maps it to an associated UniProt accession, and creates a diagram representing the UniProt protein with mapped Pfam domains): https://doi.org/10.5281/zenodo.17360009.
